# SARS-CoV-2 Infection Severity Is Linked to Superior Humoral Immunity against the Spike

**DOI:** 10.1128/mBio.02940-20

**Published:** 2021-01-19

**Authors:** Jenna J. Guthmiller, Olivia Stovicek, Jiaolong Wang, Siriruk Changrob, Lei Li, Peter Halfmann, Nai-Ying Zheng, Henry Utset, Christopher T. Stamper, Haley L. Dugan, William D. Miller, Min Huang, Ya-Nan Dai, Christopher A. Nelson, Paige D. Hall, Maud Jansen, Kumaran Shanmugarajah, Jessica S. Donington, Florian Krammer, Daved H. Fremont, Andrzej Joachimiak, Yoshihiro Kawaoka, Vera Tesic, Maria Lucia Madariaga, Patrick C. Wilson

**Affiliations:** a Department of Medicine, Section of Rheumatology, University of Chicago, Chicago, Illinois, USA; b Influenza Research Institute, Department of Pathobiological Sciences, School of Veterinary Medicine, University of Wisconsin-Madison, Madison, Wisconsin, USA; c Committee on Immunology, University of Chicago, Chicago, Illinois, USA; d Department of Medicine, Section of Pulmonary and Critical Care Medicine, University of Chicago, Chicago, Illinois, USA; e Department of Pathology and Immunology and Center for Structural Genomics of Infectious Diseases, Consortium for Advanced Science and Engineering, Washington University School of Medicine, St. Louis, Missouri, USA; f Department of Medicine, University of Chicago, Chicago, Illinois, USA; g Department of Surgery, University of Chicago, Chicago, Illinois, USA; h Department of Microbiology, Icahn School of Medicine at Mount Sinai, New York, New York, USA; i Center for Structural Genomics of Infectious Diseases, Consortium for Advanced Science and Engineering, University of Chicago, Chicago, Illinois, USA; j Structural Biology Center, X-ray Science Division, Argonne National Laboratory, Argonne, Illinois, USA; k Department of Pathology, University of Chicago, Chicago, Illinois, USA; St. Jude Children's Research Hospital

**Keywords:** SARS-CoV-2, humoral immunity, infection severity, memory B cells, neutralizing antibodies

## Abstract

Severe acute respiratory syndrome coronavirus 2 (SARS-CoV-2) is currently causing a global pandemic. The antigen specificity of the antibody response mounted against this novel virus is not understood in detail. Here, we report that subjects with a more severe SARS-CoV-2 infection exhibit a larger antibody response against the spike and nucleocapsid protein and epitope spreading to subdominant viral antigens, such as open reading frame 8 and nonstructural proteins. Subjects with a greater antibody response mounted a larger memory B cell response against the spike, but not the nucleocapsid protein. Additionally, we revealed that antibodies against the spike are still capable of binding the D614G spike mutant and cross-react with the SARS-CoV-1 receptor binding domain. Together, this study reveals that subjects with a more severe SARS-CoV-2 infection exhibit a greater overall antibody response to the spike and nucleocapsid protein and a larger memory B cell response against the spike.

## Abstract (precis)

With the ongoing pandemic, it is critical to understand how natural immunity against SARS-CoV-2 and COVID-19 develops. We have identified that subjects with more severe COVID-19 disease mount a more robust and neutralizing antibody response against SARS-CoV-2 spike protein.

## INTRODUCTION

Entry of severe acute respiratory syndrome coronavirus 2 (SARS-CoV-2) into host cells is mediated by surface trimeric spike protein via interaction between the spike receptor-binding domain (RBD) and angiotensin-converting enzyme 2 (1, 2). SARS-CoV-2 expresses numerous potential antigens, including four structural proteins (spike, nucleocapsid [N] protein, matrix, and envelope protein), 16 nonstructural proteins/antigens (NSP1 to NSP16), and several accessory open reading frame (ORF) proteins, including ORF7 and ORF8 (3, 4). Although our understanding of the specificity of the antibody response against SARS-CoV-2 is rapidly expanding (5, 6), little is known about how coronavirus disease 2019 (COVID-19) severity relates to magnitude of both the secreted antibody and memory B cell (MBC) response.

To address these critically important knowledge gaps, we collected plasma samples from SARS-CoV-2 acutely infected and convalescent subjects (7) and examined specificity of the humoral immune response. Together, our data indicate that subjects predominantly mount an antibody response against the viral spike and N protein. Moreover, our data indicate that subjects with more severe disease mount a larger antibody response, which corresponds with increased neutralizing antibody titers, MBC formation against the spike protein, and cross-reactive antibodies against conserved epitopes of the RBD. Together, our study indicates that the magnitude of the humoral immune response is related to infection severity.

## RESULTS

### SARS-CoV-2 acutely infected and convalescent subjects largely mount antibody responses against spike and N protein.

**FIG 1:**
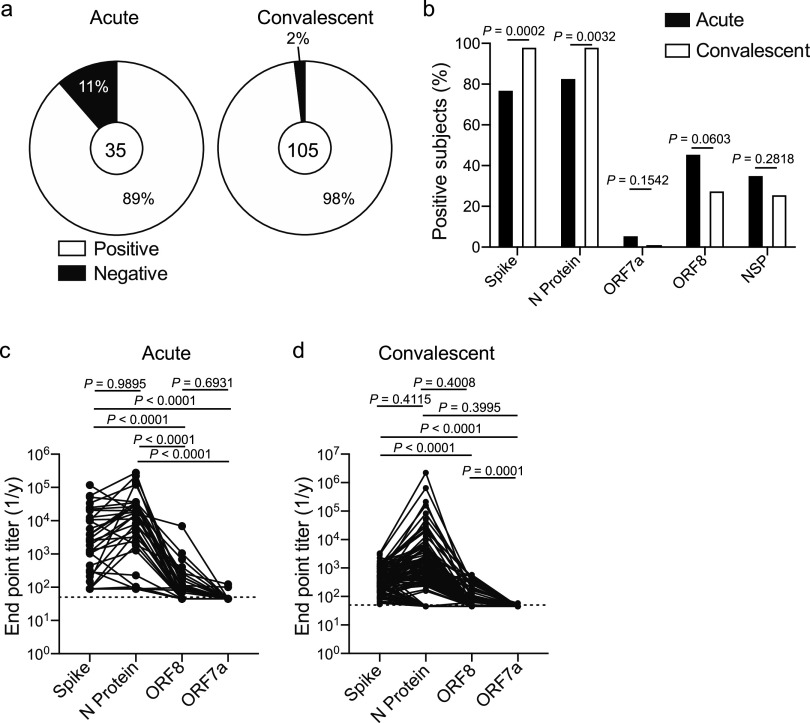
Antibody specificity and kinetics in SARS-CoV-2-infected subjects. (a) Proportion of subjects in the acutely infected and convalescent cohorts who had detectable antibodies (total Ig) to one or more SARS-CoV-2 antigens. Number in center represents the number of subjects tested in each cohort. (b) Proportion of subjects in the acutely infected (*n* = 35) and convalescent (*n* = 105) cohorts with total Ig binding spike, N protein, ORF7a, ORF8, or at least one NSP antigen. (c and d) Total Ig endpoint titers of antibodies targeting spike, N protein, ORF7a, and ORF8 in the acutely infected cohort (c) (*n* = 35) and convalescent cohort (d) (*n* = 105). Lines connect titers across one subject. Data in panel b were analyzed using Fisher’s exact tests for statistical analyses. Data in panels c and d were analyzed using paired nonparametric Friedman tests. Dashed lines in panels c and d are the limit of detection.

To address the specificity and kinetics of the humoral immune response against SARS-CoV-2, we collected plasma from 35 SARS-CoV-2-infected and 105 convalescent subjects (see Tables S1 and S2 in the supplemental material). All subjects within the acutely infected cohort were hospitalized (Table S1), and samples were collected as residual samples from the Clinical Immunology Laboratory at the University of Chicago Medical Center. Notably, 17% of subjects (6/35) had secondary viral and bacterial infections, with the one subject with secondary bacterial pneumonia being the only subject within our cohorts to succumb to COVID-19 (Table S1). The convalescent cohort were recruited to donate plasma for a convalescent plasma transfusion study at the University of Chicago Medical Center (7), and only 8% (8/105) of subjects in the convalescent cohort had been hospitalized (Table S2). Plasma from all subjects was tested against the spike, N protein, ORF7a, ORF8, and NSP3, NSP9, NSP10, and NSP15 of SARS-CoV-2. Spike and N protein are structurally important for SARS-CoV-2 and are abundantly expressed (8). ORF7a and ORF8 have immunoregulatory functions (9–11); therefore, we were interested in understanding whether subjects mounted a response against these proteins. Additionally, it has been reported that subjects mounted a large T cell response against NSP antigens (8), and NSPs may act as major antigenic targets of the humoral immune response. To detect antigen-specific antibodies, we determined seroconversion and endpoint titers using an ELISA that detects all serum antibody isotypes and subclasses. Eighty-nine percent of acutely infected subjects and 98% of convalescent subjects had detectable antibodies against one or more SARS-CoV-2 antigens (Fig. 1a), with nearly all subjects mounting a response against the spike and N protein (Fig. 1b). We further identified that convalescent subjects mounted a predominant response against the RBD of the spike protein and the RNA-binding domain of the N protein (Fig. S1a and b), suggesting these domains contain the immunodominant epitopes of these antigens. A larger frequency of acutely infected subjects mounted antibodies against ORF7a, ORF8, and NSP antigens (Fig. 1b and Fig. S1c), although these differences were not statistically significant as the acutely infected cohort size was not large enough to detect subtle differences in serum antibody specificity. These data indicate that the antibody responses at acute and convalescent time points largely target the same SARS-CoV-2 antigens.

10.1128/mBio.02940-20.1 FIG S1 Specificity of serum antibody response of SARS-CoV-2 acutely infected and convalescent subjects. (a and b) Total Ig endpoint titers of spike and RBD (a) and full-length N protein and RNA binding domain of N protein (b) from convalescent subjects (*n* = 105). Lines connect titers from the same subject. (c) Proportion of subjects with detectable antibodies (total Ig) against NSP antigens from the acute (*n* = 35) and convalescent (*n* = 105) cohorts. (d and e) Correlation of anti-spike IgG and anti-N protein IgG titers in acute (d) and convalescent (e) cohorts. Data in panels a and b were analyzed using two-tailed paired *t* tests, and data in panel c were analyzed using Fisher’s exact tests. Data in panel d were analyzed using a two-tailed Spearman correlation. Data in panel e were analyzed using a two-tailed Pearson correlation. Dashed lines in panels a and b are the limit of detection. Download FIG S1, DOCX file, 0.5 MB. Copyright © 2021 Guthmiller et al. 2021 Guthmiller et al. This content is distributed under the terms of the Creative Commons Attribution 4.0 International license.

10.1128/mBio.02940-20.5 TABLE S1 Subject and clinical information for acutely infected cohort. *Subject died as a result of COVID-19. Download Table S1, DOCX file, 0.02 MB. Copyright © 2021 Guthmiller et al. 2021 Guthmiller et al. This content is distributed under the terms of the Creative Commons Attribution 4.0 International license.

10.1128/mBio.02940-20.6 TABLE S2 Subject and clinical information for convalescent cohort. Download Table S2, DOCX file, 0.03 MB. Copyright © 2021 Guthmiller et al. 2021 Guthmiller et al. This content is distributed under the terms of the Creative Commons Attribution 4.0 International license.

We identified a strong positive correlation between the anti-N protein and anti-spike IgG titers in both the acutely infected and convalescent cohorts (Fig. S1d and e), indicating subjects who generally mounted a robust antibody response upon SARS-CoV-2 infection tended to mount a robust response against both antigens. We did not observe a statistical difference in antibody titers against the spike and N protein by individual subjects in either the acute or convalescent subject cohorts (Fig. 1c and d), likely due to dramatic subject-to-subject variation. However, antibody titers against the spike were significantly higher than antibody titers against ORF7a and ORF8 (Fig. 1c and d). Together, these data reveal the antibody response against SARS-CoV-2 is largely driven against the spike and N protein. The spike is likely an immunodominant antigen as it is the main surface glycoprotein. Although N protein is internally located within a virion, N protein completely covers the entire viral genome, likely leading to its immunodominance.

### Analysis of the humoral immune response within the acutely infected cohort.

**FIG 2:**
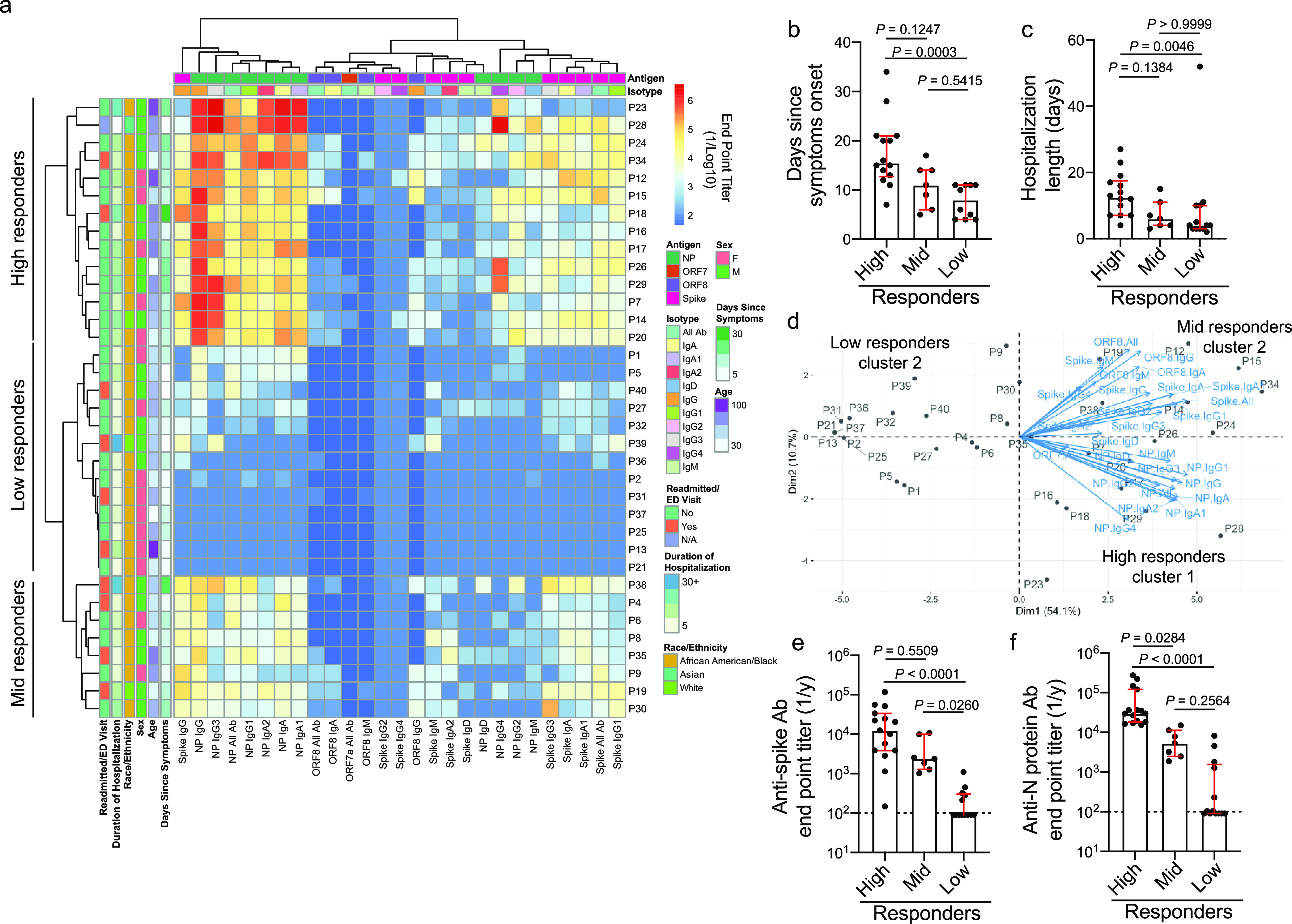
Acutely infected subjects with longer hospitalizations have a higher antibody response against N protein. (a) Heatmap of hierarchical clustering of acutely infected subjects (*n* = 35) based on antibody binding specificity and antibody isotype/subclass. Subjects clustered into three distinct clusters: high (*n* = 15), mid (*n* = 7), and low (*n* = 13) responders. (b and c) Days since symptom onset (high, *n* = 14; mid, *n* = 7; low, *n* = 10) (b) and length of hospitalization (c) among subjects in the high, mid, and low responder clusters. (d) PCA biplot of subjects clustering based on distinct antibody binding features. (e and f) Total Ig antibody titers against the spike (e) and N protein (f) among the high, mid, and low responder clusters. Data in panels b, c, e, and f were analyzed using unpaired nonparametric Kruskal-Wallis tests. Dashed lines in panels e and f are the limit of detection. Bars in panels b, c, e, and f represent the median. Unless noted otherwise (b), number of subjects per responder group is the following: high (*n* = 15), mid (*n* = 7), and low (*n* = 13) responders. Data in panels b, c, e, and f are presented as the median with interquartile range.

To understand the intersubject variability within our cohorts, we performed hierarchical clustering of subjects based on antibody titers against the spike, full length, and RNA-binding domain of N protein, ORF7a, and ORF8 antigens. We additionally tested for distinct antigen-specific antibody isotypes and subclasses. From the acutely infected cohort, we identified three clusters: high, middle (mid), and low responders (Fig. 2a and Table S3). Notably, the high responder cluster subjects were further from the onset of symptoms at the time of sampling and ultimately were hospitalized for a longer duration than those in the mid and low responder groups (Fig. 2b and c). We did not observe a statistical difference in age or sex between the three responder groups (Fig. S2a and b). Over 25% of subjects in the high responder group had a severe/highest CURB-65 score (Fig. S2c), a measure of pneumonia severity (12), suggesting subjects in the high responder group had more severe infections. Despite trends, we did not detect statistically significant differences in age, sex, and disease severity of subjects segregating into the three responder clusters, likely due to the cohort being too small. We further examined which features of the humoral immune response were driving subjects to segregate into these three clusters. Subjects within the high and mid responder groups robustly induced antibodies against the spike protein, but the high responder subjects mounted a larger response to N protein than did the mid responder subjects (Fig. 2d to f). Additionally, subjects within the high and mid responder groups were more likely to mount an antibody response against ORF8 and NSP antigens (Fig. S2d and e). The low responder group largely did not mount an antibody response against any of the antigens tested (Fig. 2d to f and Fig. S2d and e), although it is possible that plasma was collected before the subjects mounted a significant antibody response. Our data reveal that acutely infected subjects who were hospitalized for a longer duration mounted a larger antibody response against N protein and were more likely to mount a response against other SARS-CoV-2 antigens.

10.1128/mBio.02940-20.2 FIG S2 Clinical data and antibody specificity of acutely infected subject clusters. (a to c) Age (a), sex (b), and CURB-65 score (c) of subjects in the high (*n* = 15), mid (*n* = 7), and low (*n* = 13) responder clusters. (d) Total Ig endpoint titers against ORF8 of subjects in the high (*n* = 15), mid (*n* = 7), and low (*n* = 13) responder clusters. (e) Proportion of subjects in the high (*n* = 15), mid (*n* = 7), and low (*n* = 13) responder clusters with detectable antibodies (total Ig) against 1 or more NSP antigens. For panels a and c, data were analyzed by unpaired nonparametric Kruskal-Wallis tests. Data in panels b and e were analyzed by Fisher’s exact tests. Dashed lines in panel d are the limit of detection. Bars in panels a and d represent the median. Data in panels a and d are presented as the median with interquartile range. Download FIG S2, DOCX file, 0.4 MB. Copyright © 2021 Guthmiller et al. 2021 Guthmiller et al. This content is distributed under the terms of the Creative Commons Attribution 4.0 International license.

10.1128/mBio.02940-20.7 TABLE S3 *P* values between responder groups from the acutely infected cohort. Related to Fig. 2. *P* values of *post hoc* pairwise comparisons of Analysis of Variance (ANOVA) between responder groups from the acutely infected cohort. *P* values were adjusted by Holm-Bonferroni method. *P* value = 0.00000 indicates *P* value < 0.00001. Red-highlighted values represent statistically significant differences (*P* ≤ 0.05) between groups. Download Table S3, DOCX file, 0.02 MB. Copyright © 2021 Guthmiller et al. 2021 Guthmiller et al. This content is distributed under the terms of the Creative Commons Attribution 4.0 International license.

### Convalescent subjects with more severe disease mount a greater antibody response.

**FIG 3:**
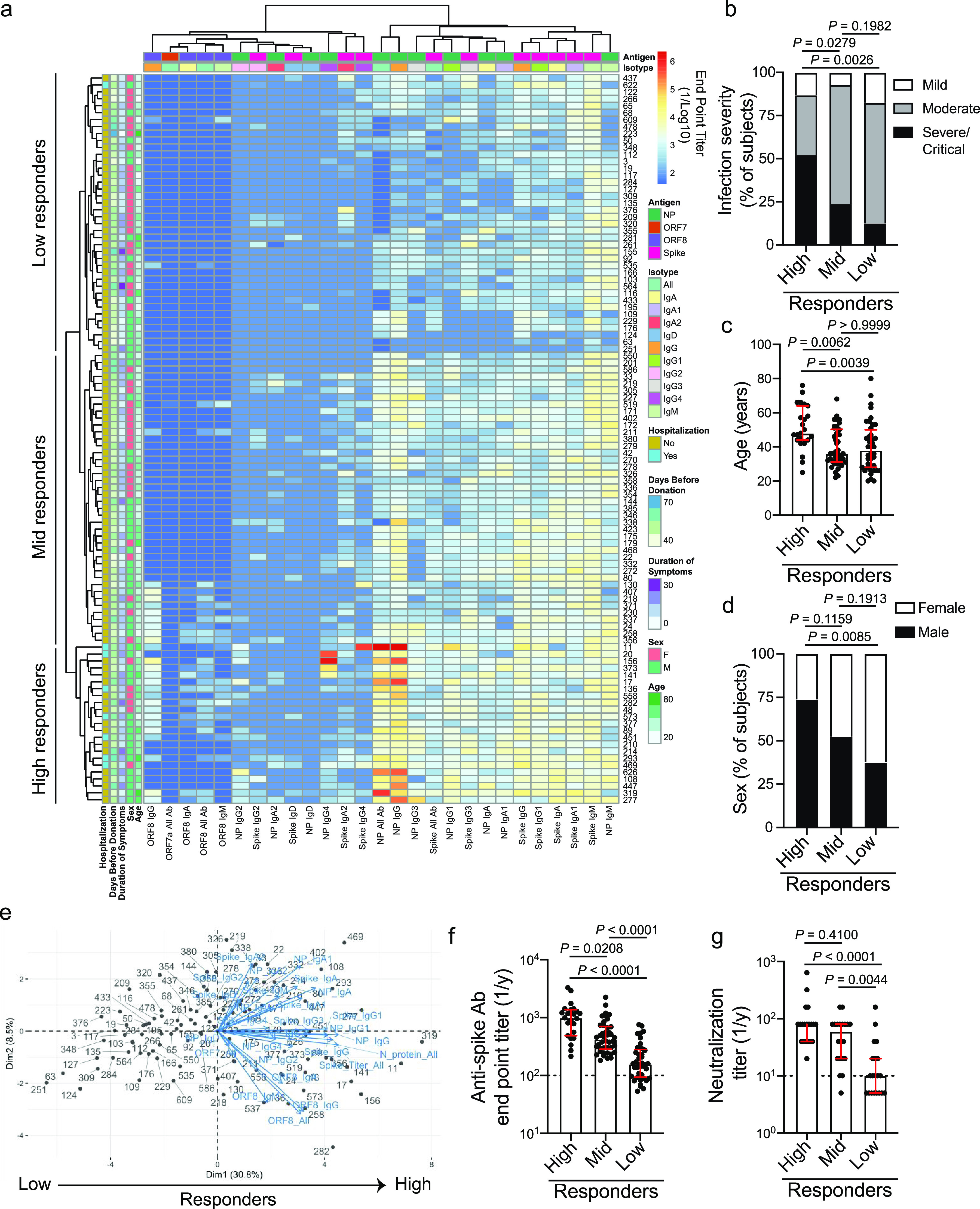
Convalescent subjects with higher antibody responses against multiple SARS-CoV-2 antigens tended to have a more severe infection. (a) Heatmap of hierarchical clustering of convalescent subjects (*n* = 105) based on antibody binding specificity and antibody isotype/subclass. Subjects clustered into three distinct clusters: high (*n* = 23), mid (*n* = 42), and low (*n* = 40) responders. (b to d) Infection severity (b), age (c), and sex (d) of subjects in the high, mid, and low responder clusters. (e) PCA biplot of subjects clustering based on distinct antibody binding features. (f) Total Ig antibody titers against the spike among the high, mid, and low responder clusters. (g) Neutralization titer, as determined by viral cytopathic effect, of 20 randomly selected samples from each of the high, mid, and low responder clusters. Data in panels f and g were analyzed using unpaired nonparametric Kruskal-Wallis tests. For panels b to d, data were analyzed using Fisher’s exact tests. Dashed lines in panels f and g are the limit of detection. Bars in panels f and g represent the median. Data in panels c, f, and g are presented as the median with interquartile range.

Using hierarchical clustering of subjects based on antibody titers against the spike, full length, and RNA-binding domain of N protein, ORF7a, and ORF8 antigens, the convalescent cohort also clustered into three distinct clusters based on the magnitude of the antibody response against the spike and N protein (Fig. 3a and Table S4), similar to the acutely infected cohort (Fig. 2). To understand the relationship between infection severity and antibody responses within the convalescent cohort, we scored subjects based on the severity and duration of self-reported symptoms and whether subjects were hospitalized (Table S5). Notably, over 50% of subjects within the high responder group had a severe infection (Fig. 3b and Fig. S3a), indicating infection severity is linked to increased antibody titers. Moreover, subjects within the high responder group typically were older and male (Fig. 3c and d), and 7/8 subjects who were hospitalized with COVID-19 fell within the high response cluster (Table S2). Subjects within each responder group had a similar duration of symptoms (Fig. S3b), and subjects within all three groups had a similar amount of time to mount a response, as determined by the number of days since symptom onset at the time of donation (Fig. S3c).

10.1128/mBio.02940-20.3 FIG S3 Clinical data and antibody specificity of convalescent subject clusters. (a to c) Severity score (a), duration of symptoms (b), and days since symptom onset (c) of subjects in the high (*n* = 23), mid (*n* = 42), and low (*n* = 40) responder clusters. (d and e) Total Ig endpoint titers against N protein (d) and ORF8 (e) of subjects in the high (*n* = 23), mid (*n* = 42), and low (*n* = 40) responder clusters. (f) Proportion of subjects in the high (*n* = 23), mid (*n* = 42), and low (*n* = 40) responder clusters with detectable antibodies (total Ig) against 1 or more NSP antigens. For panels a to e, data were analyzed by unpaired nonparametric Kruskal-Wallis tests. Data in panel f were analyzed by Fisher’s exact tests. Dashed lines in panels d and e are the limit of detection. Bars in panels a to e represent the median. Data in panels a to e are presented as the median with interquartile range. Download FIG S3, DOCX file, 0.5 MB. Copyright © 2021 Guthmiller et al. 2021 Guthmiller et al. This content is distributed under the terms of the Creative Commons Attribution 4.0 International license.

10.1128/mBio.02940-20.8 TABLE S4 *P* values between responder groups from the convalescent cohort. Related to Fig. 3. *P* values of *post hoc* pairwise comparisons of Analysis of Variance (ANOVA) between responder groups from the convalescent cohort. *P* values were adjusted by Holm-Bonferroni method. *P* value = 0.00000 indicates *P* value < 0.00001. Red-highlighted values represent statistically significant differences (*P* ≤ 0.05) between groups. Download Table S4, DOCX file, 0.01 MB. Copyright © 2021 Guthmiller et al. 2021 Guthmiller et al. This content is distributed under the terms of the Creative Commons Attribution 4.0 International license.

10.1128/mBio.02940-20.9 TABLE S5 Infection severity scoring system for convalescent subjects based on symptoms, hospitalization, and duration of symptoms. Download Table S5, DOCX file, 0.01 MB. Copyright © 2021 Guthmiller et al. 2021 Guthmiller et al. This content is distributed under the terms of the Creative Commons Attribution 4.0 International license.

Unlike the acutely infected cohort, subjects within the high responder group had higher titers against not only the N protein but also the spike and ORF8 antigens relative to subjects within the mid and low responder groups (Fig. 3e and f and Fig. S3d and e) and were trending to be more likely to seroconvert against at least one of the NSP antigens tested (Fig. S3f). Furthermore, subjects in the high responder cluster mounted a higher IgG1 and IgG3 antibody response against the spike (Table S4). IgG1 and IgG3 antibody subclasses are classically associated with neutralization, complement activation, and Fc-mediated effector functions (13), key features of protective humoral immune responses against viruses. Consistent with these data, high and mid responder subjects had higher neutralizing titers than subjects in the low responder cohorts (Fig. 3g). Additionally, anti-N protein IgG4 was one of the main driving factors leading to segregation of the three responder clusters (Table S4). As IgG4 is classically associated with sclerosing and fibrotic diseases (14), increased pneumonia severity and lung fibrosis could lead to isotype class switching to this relatively rare subclass. Additionally, we identified that nearly all subjects had persistent IgM antibody responses against the spike and N protein (Fig. 3a), suggesting IgM persists into convalescence. Altogether, our data reveal subjects with more severe infection are mounting a larger antibody response at convalescent time points.

### MBCs largely target the spike and correlate with secreted antibody titers.

**FIG 4:**
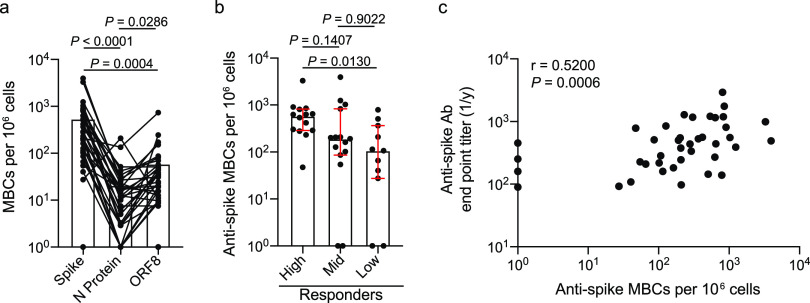
MBC response is largely driven against the spike. (a and b) PBMCs from convalescent donors were polyclonally stimulated, and ELISpots were performed to assess the number of antigen-specific MBCs. (a) Number of IgG/IgA^+^ MBCs (antigen-specific MBCs per 10^6^ cells) targeting the spike, N protein, or ORF8 (*n* = 36). Lines connect antigen-specific MBCs across subjects. (b) Number of spike-targeting IgG/IgA^+^ MBCs among the high (*n* = 14), mid (*n* = 15), and low (*n* = 11) responder clusters. (c) Spearman correlation of the number of anti-spike IgG/IgA^+^ MBCs and anti-spike endpoint titers by individual (*n* = 40). Data in panel a were analyzed using paired nonparametric Friedman tests. Data in panel b were analyzed using unpaired nonparametric Kruskal-Wallis tests. Data in panel c were analyzed by a nonparametric two-tailed Spearman correlation. Data in panel b are presented as the median with interquartile range.

We next dissected the specificities of MBCs induced by SARS-CoV-2 infection by performing B cell ELISpots on polyclonally stimulated peripheral blood mononuclear cells (PBMCs) isolated from convalescent subjects, a common technique used to probe class-switched MBC specificities (15). Notably, we focused our studies on IgG and IgA class-switched MBCs to avoid background of low-affinity IgM-secreting cells. MBCs largely targeted the spike, whereas very few MBCs targeted N protein or ORF8 (Fig. 4a). Additionally, subjects in the serum high responder group mounted a larger MBC response against the spike than subjects in the mid and low responder cohorts (Fig. 4b), with serum antibody titers against the spike positively correlating with the magnitude of the anti-spike MBC response (Fig. 4c). Despite the observed differences in anti-spike MBC responses between responder groups, we did not observe any differences in the anti-N protein and anti-ORF8 MBC response in the three responder cohorts (Fig. S4a and b). Together, these data indicate that the MBC response is largely directed against the spike protein and that the high serum responder group mounted both a larger secreted antibody and larger MBC response upon SARS-CoV-2 infection.

10.1128/mBio.02940-20.4 FIG S4 MBC responses against N protein and ORF8. (a and b) PBMCs from convalescent donors were polyclonally stimulated, and ELISpots were performed to assess the number of antigen-specific MBCs. (a) Number of IgG/IgA^+^ MBCs (antigen-specific MBCs per 10^6^ cells) targeting N protein (a) and ORF8 (b) among the high (*n* = 14 for N protein; *n* = 11 for ORF8), mid (*n* = 16 for N protein; *n* = 15 for ORF8), and low (*n* = 10 for N protein and ORF8) responder clusters. For panels a and b, data were analyzed by unpaired nonparametric Kruskal-Wallis tests. Data in are presented as the median with interquartile range. Download FIG S4, DOCX file, 0.4 MB. Copyright © 2021 Guthmiller et al. 2021 Guthmiller et al. This content is distributed under the terms of the Creative Commons Attribution 4.0 International license.

### Spike antibodies cross-react with the D614G mutant and SARS-CoV-1.

**FIG 5:**
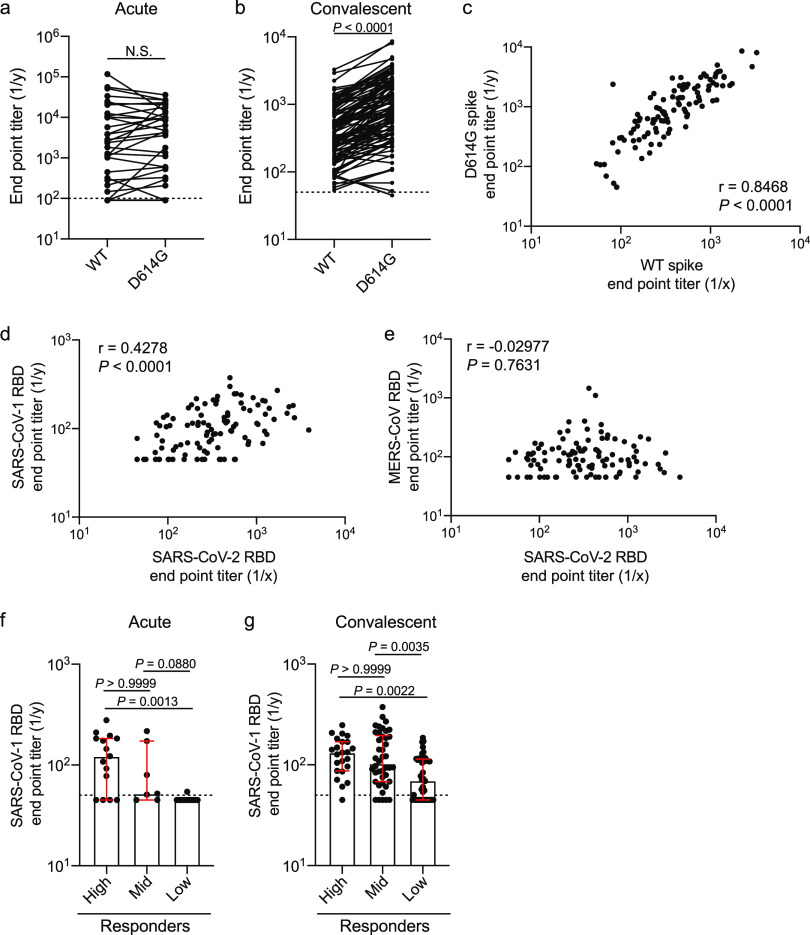
Antibody cross-reactivity to G614 spike mutant and SARS-CoV-1 and MERS-CoV RBD. (a and b) Endpoint titers of total Ig antibodies binding to the WT (D614) and mutant (D614G) SARS-CoV-2 spike protein from the acute (a) (*n* = 35) and convalescent (b) (*n* = 105) cohorts. (c) Correlation of total Ig endpoint titers against the WT (D614) and mutant (D614G) spike from the convalescent cohort (*n* = 105). (d and e) Correlation between SARS-CoV-2 RBD total Ig endpoint titers and SARS-CoV-1 RBD (d) or MERS-CoV RBD (e) total Ig endpoint titers from convalescent subjects (*n* = 105). (f and g) SARS-CoV-1 RBD total Ig endpoint titers among the high, mid, and low responder clusters from the acutely infected cohort (f) (high, *n* = 23; mid, *n* = 42; and low, *n* = 40) and the convalescent cohort (g) (high, *n* = 23; mid, *n* = 42; and low, *n* = 40). Data in panel a were analyzed using a two-tailed Wilcoxon matched-pairs signed-rank test. Data in panel b were analyzed using a two-tailed paired *t* test. For panels c to e, data were analyzed using a two-tailed Pearson correlation. Data in panels f and g were analyzed using unpaired nonparametric Kruskal-Wallis tests. Dashed lines in panels a, b, f, and g are the limit of detection. Bars in panels f and g represent the median. Data in panels f and g are presented as the median with interquartile range.

SARS-CoV-2 has acquired a D614G mutation within the spike protein, and viruses carrying this mutation have since become the dominant circulating strain globally as of early April 2020 (16). This mutation is located on the interface between two subunits of the spike trimer and may impact stability of the trimer (1). As the subjects within our study were initially infected throughout March and into early April 2020 (Tables S1 and 2), they were likely infected with the D614 variant. We did not observe a difference in antibody titers against the wild-type (WT) and D614G spike antigens within our acute cohort (Fig. 5a), suggesting the D614G epitope was not a major antigenic site. Strikingly, we identified that the convalescent cohort mounted a larger response against the G614 variant than against the WT D614 that they were likely infected with (Fig. 5b), potentially due to the increased stability of the G614 variant (17). Furthermore, we observed a strong positive correlation between D614 (WT) spike titers and G614 titers, indicating antibodies against the WT strain likely protect against the new G614 variant (Fig. 5c). These data indicate that the region that encompasses the D614G mutation is not immunodominant or does not affect the antigenicity of epitopes at or near this site. We also examined whether antibodies targeting the RBD of the spike protein cross-reacted with the RBD proteins of other pandemic threat coronaviruses, including SARS-CoV-1 and Middle East respiratory syndrome (MERS) CoV. We found a positive correlation between antibody titers against the SARS-CoV-2 RBD and the SARS-CoV-1 RBD, but not the MERS-CoV RBD (Fig. 5d and e). When divided by responder groups (Fig. 2 and 3), subjects in the high and mid responder groups had elevated titers against the SARS-CoV-1 RBD (Fig. 5f and g). These data show that subjects who mounted a larger response against the SARS-CoV-2 spike protein additionally mounted a larger antibody response against conserved epitopes that cross-react with closely related coronaviruses.

## DISCUSSION

Together, our study demonstrates that severity of SARS-CoV-2 infection is associated with an increase in the magnitude and breadth of the ensuing humoral immune response. Notably, we identified that the antibody response is largely mounted against the spike and N proteins. Although both proteins are highly expressed by coronaviruses, there is much more N protein as it encapsulates the whole viral genomic RNA, which is nearly 30 kb in size. As N protein dimer is projected to bind about 30 bp (18), there are likely 1,000+ N proteins per virion. In sharp contrast, there are only ∼26 spike trimers per virion (19), suggesting the immunodominance toward N protein may be related to antigen burdens. Likewise, subjects with more severe disease likely have increased viral titers and free antigen in the lung lumen and draining lymph nodes, which could lead to increased antibody titers against nearly all antigens tested. Therefore, epitope spreading of the antibody response may be a factor of the amount of SARS-CoV-2 antigen present.

Subjects also mounted an antibody response against the accessory protein ORF8, which has immunoregulatory properties including the ability to limit type I interferon responses (11, 20) and downregulate MHC-I presentation to CD8 T cells (9). ORF8 is found in the serum of COVID-19 subjects (21), suggesting antibodies targeting ORF8 may limit these immunoregulatory properties, improve the host immune response, and achieve better clinical disease outcomes. However, whether and how antibodies against ORF8 mediate protection are yet to be determined. Additionally, we identified antibodies against nonstructural proteins involved in viral replication, although antibodies against these antigens are unlikely to provide protection, as these antibodies targeting NSPs would need to be inside a live cell while virus is replicating. Whether antibodies targeting discrete viral antigens other than the spike are neutralizing, have Fc-mediated effector functions, or are protective during infection is yet to be determined.

Our study revealed that acutely infected subjects who mounted higher antibody response relative to mid and low responder clusters tended to have higher pneumonia severity scores. Consistent with this notion, convalescent subjects who had higher antibody titers were those subjects who had a more severe infection. A recent report identified that subjects who succumbed to COVID-19 tended to mount a larger antibody response against N protein relative to the spike, whereas convalescent subjects tended to focus their antibody response on the spike protein (22). However, our study and others (5, 6) have identified that subjects generally had similar antibody responses against the N protein and spike. Additionally, our study reveals infection severity was linked to an increase in antibody responses against both the spike and N protein. Ultimately, our findings on the relationship between infection severity and increased titers against the spike are consistent with a recent surveillance study performed in Iceland (23).

The best clinical predictors of the magnitude of the antibody responses and epitope spreading within our convalescent cohort were age, sex, and hospitalization, which is consistent with other reports (24). The median age of the high responder cluster was 10+ years greater than those for the mid and low responder clusters (48 years versus 36 and 38 years, respectively). Older adults are more likely to be symptomatic and hospitalized with SARS-CoV-2 infection (25, 26), suggesting increased disease severity and sustained viral titers over a longer period of time could lead to greater antibody titers against multiple viral antigens. Similarly, males were more likely to be segregated into the higher responder group despite the common finding that females generally mount higher antibody responses upon other viral infections and upon vaccination (27). Although there is no difference in incidence of COVID-19 in men and women, men have a higher morbidity and mortality rate than women (28, 29) and likely experience increased viral titers and antigen persistence. Altogether, disease severity is the main clinical predictor of the magnitude of the antibody response mounted against SARS-CoV-2, as men and older adults are more likely to be hospitalized with COVID-19. Our data also demonstrated that subjects with more severe disease tended to mount a more cross-reactive antibody response against the SARS-CoV-1 RBD, indicating subjects were mounting antibodies against conserved epitopes of the RBD. Although cross-reactive antibodies between SARS-CoV-1 and SARS-CoV-2 have previously been reported (30), our study identifies that subjects with more severe disease are more likely to drive antibodies against conserved CoV epitopes of the RBD.

Together, our data indicate more severe infection is linked to a larger magnitude of circulating antibody and MBC response and increased viral antigen binding breadth across different viral antigens. CD4 T cells are critical for driving antibody responses by mediating germinal center selection of antigen-specific B cells. Notably, CD4 T cells targeting multiple SARS-CoV-2 antigens and the magnitude of the CD4 T cell response positively correlate with SARS-CoV-2-specific antibody responses (8, 31). Moreover, subjects with more severe disease demonstrate an increased breadth and magnitude of the memory CD4 T cell response (31), which could lead to the larger and broader antibody response of subjects with more severe infection, as observed in our study. The increase in the magnitude of the antibody response and MBC response in subjects with more severe infection could be due to increased CD4 T cell responses, although this was not directly tested in our study. However, subjects who succumbed to SARS-CoV-2 infection demonstrated a loss of germinal centers and CD4 T follicular helper cells (32). Additionally, subjects with more severe disease largely mounted an extrafollicular B cell response, which could lead to B cells of lower affinity against viral antigens (33). These data in conjunction with our study suggest that an immunological balance will be needed to drive a sufficient secreted antibody response, MBC differentiation, and memory T cell responses that could provide robust protection from reinfection while preventing significant morbidity and mortality associated with SARS-CoV-2 infection.

## MATERIALS AND METHODS

### Study cohorts and sample collection.

All studies were performed with the approval of the University of Chicago institutional review board and University of Chicago and University of Wisconsin-Madison institutional biosafety committees. Plasma samples from the acutely infected cohort were collected as residual samples submitted to the University of Chicago Medicine Clinical Laboratories, and informed consent was not required. For convalescent subjects, informed consent was obtained after the research applications and possible consequences of the studies were disclosed to study subjects. This clinical trial was registered at ClinicalTrials.gov with identifier NCT04340050, and clinical information for patients included in the study is detailed in Table S2 in the supplemental material. Leukoreduction filter donors were 18 years of age or older, eligible to donate blood as per standard University of Chicago Medicine Blood Donation Center guidelines, had a documented COVID-19 PCR positive test, and had complete resolution of symptoms at least 28 days prior to donation. PBMCs were collected from leukoreduction filters within 2 h postcollection and flushed from the filters using sterile 1× phosphate-buffered saline (PBS; Gibco) supplemented with 0.2% bovine serum albumin (BSA; Sigma). Lymphocytes were purified by Lymphoprep Ficoll gradient (Thermo Fisher), and contaminating red blood cells were lysed by ACK buffer (Thermo Fisher). Cells were frozen in fetal bovine serum (FBS; Gibco) with 10% dimethyl sulfoxide (DMSO; Sigma) prior to downstream analysis. Three milliliters of whole blood in sodium citrate tubes was obtained for plasma collection. All subjects in the acute and convalescent cohorts had PCR-confirmed SARS-CoV-2 infections.

### Recombinant proteins.

Plasmids for the SARS-CoV-2 RBD and spike were provided by Florian Krammer at Icahn School of Medicine at Mount Sinai, and recombinant proteins were expressed in-house in HEK293F cells. D614G spike protein, SARS-CoV-1 RBD, and MERS-CoV RBD were generated in-house and expressed in HEK293F cells. ORF7a, ORF8, and full-length N proteins were cloned from the 2019-nCoV/USA-WA1/2020 SARS-CoV-2 strain at Washington University. Proteins were expressed in Escherichia coli, with N protein purified as a soluble protein and ORF7a and ORF8 oxidatively refolded from inclusion bodies. NSP antigens and the RNA-binding domain of N protein were provided by Andrzej Joachimiak at the Center for Structural Genomics of Infectious Diseases at the University of Chicago and Argonne National Laboratory and were expressed in Escherichia coli.

### Enzyme-linked immunosorbent assay (ELISA).

ELISAs performed in this study were adapted from previously established protocols (34, 35). Plasma samples were heat inactivated for 1 h at 56°C. High protein-binding microtiter plates (Costar) were coated with recombinant antigens at 2 μg/ml in phosphate-buffered saline (PBS) overnight at 4°C. Plates were washed with PBS-0.05% Tween and blocked with 200 μl PBS-0.1% Tween plus 3% milk powder for 1 h at room temperature. Plasma samples were serially diluted in PBS-0.1% Tween plus 1% milk powder. Plates were incubated with serum dilutions for 2 h at room temperature. Horseradish peroxidase (HRP)-conjugated goat anti-human Ig secondary antibody diluted in PBS-0.1% Tween plus 1% milk powder was used to detect binding of antibodies, and after a 1-h incubation, plates were developed with 100 μl SigmaFast OPD solution (Sigma-Aldrich), with development reaction stopped after 10 min using 50 μl 3 M HCl. Absorbance was measured at 490 nm on a microplate spectrophotometer (Bio-Rad). To detect binding of specific antibody isotypes and subclasses, ELISAs were performed using alternate secondary antibodies (Sigma-Aldrich; Jackson ImmunoResearch; Southern Biotech). Endpoint titers were extrapolated from a sigmoidal 4PL (where X is log concentration) standard curve for each sample. Limit of detection (LOD) is defined as the mean plus 3 SD of the optical density (OD) signal recorded using plasma from SARS-CoV-2-negative human subjects. All calculations were performed in Prism 8 (GraphPad).

### Neutralization assays.

Neutralization assays were performed by a viral cytopathic effect (CPE) assay using the SARS-CoV-2/UW-001/Human/2020/Wisconsin (UW-001) virus, which was isolated from a mild human case in Wisconsin. Plasma was diluted 1:5, serially diluted 2-fold, and mixed with an equal volume of virus (100 PFU) for a starting dilution of 1:10. The plasma-virus mixture was incubated for 30 min at 37°C and added to TMPRSS2-expressing Vero E6 cells grown in 1× minimum essential medium (MEM) supplemented with 5% fetal calf serum (FCS). Cells were incubated with plasma-virus mixture for 3 days and then fixed, stained, and analyzed. CPE was observed under an inverted microscope, and neutralization titers were determined as the highest serum dilution that completely prevented CPE.

### MBC stimulations and enzyme-linked immunospot assays (ELISpot).

MBC stimulations were performed on PBMCs collected from subjects in the convalescent cohort. To induce MBC differentiation into antibody-secreting cells, 1 × 10^6^ PBMCs were stimulated with 10 ng/ml lectin pokeweed mitogen (Sigma-Aldrich), 1/100,000 protein A from Staphylococcus aureus, Cowan strain (Sigma-Aldrich), and 6 μg/ml CpG (Invitrogen) in complete RPMI in an incubator at 37°C and 5% CO_2_ for 5 days. After stimulation, cells were counted and added to ELISpot white polystyrene plates (Thermo Fisher) coated with 4 μg/ml of SARS-CoV-2 spike that were blocked with 200 μl of complete RPMI. ELISpot plates were incubated with cells for 16 h overnight in an incubator at 37°C and 5% CO_2_. After the overnight incubation, plates were washed and incubated with anti-IgG-biotin and anti-IgA-biotin (Mabtech) for 2 h at room temperature. After secondary antibody incubation, plates were washed and incubated with streptavidin-alkaline phosphatase (Southern Biotech) for 2 h at room temperature. Plates were washed and developed with NBT/BCIP (Thermo Fisher Scientific) for 2 to 10 min, and reactions were stopped by washing plates with distilled water and allowing the plates to dry overnight before counting. Images were captured with Immunocapture 6.4 software (Cellular Technology Ltd.), and spots were manually counted. All data are represented as the number of antigen-specific antibody-secreting cells per 10^6^ live cells counted after 5 days.

### Infection severity scoring and CURB-65 scoring.

For the acutely infected cohort, CURB-65 (12) scores were calculated based on confusion, blood urea nitrate levels, respiratory rate, blood pressure, and age of subjects. For the convalescent cohort, we designed a severity scoring system (see Table S5 in the supplemental material) based on presence of 12 symptoms, duration of symptoms, and hospitalization, with a maximum of 35 points possible. Symptoms were scored based on presence or absence of 12 symptoms, severity (mild or moderate) of symptoms, with a possibility of 17 points. Duration of symptoms was broken down based on the number of weeks of symptoms. Hospitalized subjects were broken down based on oxygen supplementation and intensive care unit (ICU) admission. The criteria for scoring and the classification of certain scores (mild, moderate, severe, and critical infection) were determined before analyzing the data.

### Heatmaps, hierarchical clustering, and statistical analysis.

Heatmaps were generated by ‘pheatmap’ *R* package (version 1.0.12). Features and subjects were clustered by the hierarchical clustering method implemented in the ‘pheatmap’ *R* package. Principal-component analyses (PCA) were performed using ‘factoextra’ *R* package (version 1.0.7). Subjects were then visualized by their first two principal components (PC1 and PC2) on a 2D map. All statistical analysis was performed using Prism software (GraphPad version 8), or *R* (version 3.6.3). All data presented are distinct samples. Specific tests for statistical significance used are indicated in the corresponding figure legends. Where applicable, statistical analyses were two-sided. *P* values less than or equal to 0.05 were considered statistically significant.

### Availability of materials.

Biological samples are unique to this study. Non-commercially available materials within this study are available upon request to the corresponding authors.

### Data availability.

We declare that all data supporting the findings of this study are available within the paper and its supplemental material files.
