# Supplementary material for: SARS-CoV-2 Infection Severity Is Linked to Superior Humoral Immunity against the Spike
Source: mBio. 2021 Jan 19;12(1):e02940-20. doi: 10.1128/mBio.02940-20 (PMC7845638; doi:10.1128/mBio.02940-20)
Supplement: FIG S1 [file mBio.02940-20-sf001.docx]

**Extended data Fig. 1: Specificity of serum antibody response of SARS-CoV-2 acutely infected and convalescent subjects. a** and **b**, Total Ig end point titers of spike and RBD (**a**) and full-length N protein and RNA binding domain of N protein (**b**) from convalescent subjects (n=105). Lines connect titers from the same subject. **c**, Proportion of subjects with detectable antibodies (total Ig) against NSP antigens from the acute (n=35) and convalescent cohorts (n-105). **d** and **e**, correlation of anti-spike IgG and anti-N protein IgG titers in acute (**d**) and convalescent (**e**) cohorts. Data in **a** and **b** were analyzed using two-tailed paired t tests and data in **c** were analyzed using Fisher’s exact tests**.** Data in **d** were analyzed using a two-tailed Spearman correlation. Data in **e** were analyzed using a two-tailed Pearson correlation. Dashed lines in **a** and **b** are the limit of detection.
