# Supplementary material for: SARS-CoV-2 Infection Severity Is Linked to Superior Humoral Immunity against the Spike
Source: mBio. 2021 Jan 19;12(1):e02940-20. doi: 10.1128/mBio.02940-20 (PMC7845638; doi:10.1128/mBio.02940-20)
Supplement: TABLE S1 [file mBio.02940-20-st001.docx]

**Supplemental Table 1: Subject and clinical information for acutely infected cohort**

*Subject died as a result of COVID-19

| **Subject** | **Duration of Hospitalization (Days)** | **Days Since Symptom Onset** | **Age** | **Sex** | **Responder Cluster** | **CURB-65 Score** | **Date of Symptom Onset (MM/DD/YY)** | **Secondary Infection** |
| --- | --- | --- | --- | --- | --- | --- | --- | --- |
| P1 | 4 | 11 | 50 | F | Low | 1-Low | 3/20/20 |  |
| P2 | 3 | 4 | 39 | F | Low | 0 | 3/25/20 |  |
| P4 | 4 | 5 | 45 | M | Mid | 0 | 3/23/20 |  |
| P5 | 10 | unknown | 53 | M | Low | 1-Low | unknown |  |
| P6 | 7 | 14 | 51 | F | Mid | 0 | 3/20/20 |  |
| P7 | 7 | 13 | 66 | F | High | 1-Low | 3/20/20 |  |
| P8 | 6 | 9 | 37 | M | Mid | 0 | 3/22/20 |  |
| P9 | 4 | 6 | 72 | F | Mid | 1-Low | 3/25/20 |  |
| P12 | 12 | 14 | 88 | F | High | 1-Low | 3/25/20 |  |
| P13 | 11 | 4 | 100 | F | Low | 2-Moderate | 3/27/20 | Influenza A |
| P14 | 16 | 15 | 58 | M | High | 3-Severe | 3/26/20 |  |
| P15 | 7 | 11 | 50 | F | High | 0 | 3/23/20 |  |
| P16 | 4 | 7 | 71 | M | High | 1-Low | 3/25/20 |  |
| P17 | 14 | 16 | 79 | F | High | 3-Severe | 3/25/20 |  |
| P18 | 19 | 34 | 78 | M | High | 1-Low | 3/15/20 |  |
| P19 | 11 | 14 | 38 | M | Mid | 0 | 3/25/20 | Influenza A |
| P20 | 10 | 12 | 37 | F | High | 0 | 3/27/20 |  |
| P21 | 2 | 4 | 41 | F | Low | 0 | 3/27/20 |  |
| P23 | 23 | 21 | 90 | M | High | 2-Moderate | 3/26/20 |  |
| P24 | 12 | 20 | 67 | M | High | 2-Moderate | 3/21/20 |  |
| P25 | 3 | 11 | 67 | F | Low | 1-Low | 3/21/20 | Rhinovirus/  Enterovirus |
| P26 | 13 | 20 | 28 | M | High | 0 | 3/21/20 |  |
| P27 | 3 | 11 | 66 | F | Low | 2-Moderate | 3/21/20 |  |
| P28* | 10 | unknown | 73 | M | High | 4-Highest | unknown | *B.parapertussis* |
| P29 | 17 | 21 | 73 | M | High | 3-Severe | 3/25/20 |  |
| P30 | 15 | 17 | 65 | M | Mid | 1-Low | 3/25/20 |  |
| P31 | 3 | 5 | 66 | F | Low | 1-Low | 3/27/20 |  |
| P32 | 4 | 6 | 52 | M | Low | 0 | 3/25/20 |  |
| P34 | 7 | 15 | 34 | M | High | 0 | 3/21/20 |  |
| P35 | 3 | 11 | 80 | M | Mid | 1-Low | 3/21/20 |  |
| P36 | 4 | 11 | 61 | M | Low | 1-Low | 3/21/20 |  |
| P37 | 5 | 10 | 30 | F | Low | 0 | 3/21/20 |  |
| P38 | 27 | 28 | 57 | M | High | 1-Low | 4/10/20 |  |
| P39 | 52 | unknown | 57 | M | Low | 0 | unknown | RSV; Coronavirus HKU1 |
| P40 | 10 | unknown | 63 | M | Low | 2-Moderate | unknown | Rhinovirus/Enterovirus |
