# Supplementary material for: SARS-CoV-2 Infection Severity Is Linked to Superior Humoral Immunity against the Spike
Source: mBio. 2021 Jan 19;12(1):e02940-20. doi: 10.1128/mBio.02940-20 (PMC7845638; doi:10.1128/mBio.02940-20)
Supplement: TABLE S2 [file mBio.02940-20-st002.docx]

**Supplemental Table 2: Subject and clinical information for convalescent cohort**

| **Donor** | **Duration symptoms (Days)** | **Time from symptom start to donation (Days)** | **Hospitalized** | **Age** | **Sex** | **Responder Cluster** | **Infection Severity Score** | **Infection Severity Category** | **SARS-CoV-2 PCR Test Date (MM/DD/YY)** |
| --- | --- | --- | --- | --- | --- | --- | --- | --- | --- |
| 63 | 2 | 33 | No | 44 | M | Low | 12 | Moderate | 3/30/20 |
| 109 | 9 | 41 | No | 34 | M | Low | 14 | Moderate | 3/15/20 |
| 229 | 2 | 42 | No | 55 | M | Low | 4 | Mild | 3/11/20 |
| 309 | 6 | 36 | No | 29 | F | Low | 16 | Moderate | 3/17/20 |
| 176 | 6 | 35 | No | 26 | M | Low | 11 | Moderate | 3/22/20 |
| 3 | 4 | 33 | No | 20 | M | Low | 12 | Moderate | 3/16/20 |
| 42 | 11 | 39 | No | 30 | M | Mid | 11 | Moderate | 3/18/20 |
| 112 | 9 | 40 | No | 43 | M | Low | 16 | Moderate | 3/20/20 |
| 270 | 9 | 39 | No | 50 | M | Mid | 15 | Moderate | 3/18/20 |
| 319 | 4 | 36 | No | 76 | M | High | 10 | Mild | 3/27/20 |
| 122 | 6 | 35 | No | 26 | F | Low | 14 | Moderate | 3/20/20 |
| 218 | 19 | 48 | No | 51 | F | Mid | 19 | Severe | 3/16/20 |
| 22 | 3 | 31 | No | 31 | F | Mid | 12 | Moderate | 3/23/20 |
| 108 | 11 | 39 | No | 58 | M | High | 14 | Moderate | 3/15/20 |
| 373 | 7 | 39 | No | 48 | M | High | 11 | Moderate | 3/16/20 |
| 272 | 14 | 43 | No | 42 | M | Mid | 18 | Moderate | 3/18/20 |
| 332 | 6 | 35 | No | 32 | M | Mid | 13 | Moderate | 3/21/20 |
| 179 | 6 | 37 | No | 68 | F | Mid | 10 | Mild | 3/18/20 |
| 385 | 7 | 47 | No | 33 | M | Mid | 15 | Moderate | 3/11/20 |
| 433 | 6 | 35 | No | 33 | M | Low | 13 | Moderate | 3/20/20 |
| 377 | 9 | 41 | No | 44 | M | High | 17 | Moderate | 3/14/20 |
| 355 | 14 | 44 | No | 45 | F | Low | 17 | Moderate | 3/14/20 |
| 89 | 13 | 43 | No | 64 | M | High | 8 | Mild | 3/19/20 |
| 278 | 12 | 47 | No | 52 | F | Mid | 15 | Moderate | 3/12/20 |
| 346 | 11 | 39 | No | 30 | M | Mid | 16 | Moderate | 3/16/20 |
| 130 | 7 | 35 | No | 52 | M | Mid | 10 | Mild | 3/26/20 |
| 135 | 7 | 36 | No | 28 | F | Low | 13 | Moderate | 3/24/20 |
| 266 | 4 | 32 | No | 20 | F | Low | 9 | Mild | 3/25/20 |
| 251 | 22 | 51 | No | 53 | M | Low | 19 | Severe | 3/18/20 |
| 19 | 14 | 44 | No | 55 | F | Low | 17 | Moderate | 3/15/20 |
| 80 | 12 | 40 | No | 33 | M | Mid | 18 | Moderate | 3/26/20 |
| 117 | 15 | 46 | No | 21 | F | Low | 18 | Moderate | 3/14/20 |
| 124 | 5 | 34 | No | 45 | M | Low | 4 | Mild | 3/25/20 |
| 195 | 10 | 41 | No | 26 | F | Low | 15 | Moderate | 3/22/20 |
| 284 | 11 | 39 | No | 26 | F | Low | 16 | Moderate | 3/19/20 |
| 103 | 13 | 41 | No | 33 | M | Low | 14 | Moderate | 3/19/20 |
| 336 | 14 | 42 | No | 47 | F | Mid | 14 | Moderate | 3/19/20 |
| 227 | 14 | 44 | No | 48 | M | Mid | 18 | Moderate | 3/17/20 |
| 354 | 7 | 37 | No | 28 | F | Mid | 13 | Moderate | 4/23/20 |
| 92 | 16 | 47 | No | 36 | M | Low | 16 | Moderate | 3/14/20 |
| 141 | 19 | 48 | No | 66 | M | High | 15 | Moderate | 3/20/20 |
| 230 | 13 | 42 | No | 31 | F | Mid | 17 | Moderate | 3/21/20 |
| 24 | 12 | 41 | No | 34 | M | Mid | 19 | Severe | 3/23/20 |
| 156 | 11 | 41 | No | 50 | F | High | 14 | Moderate | 3/23/20 |
| 171 | 16 | 44 | No | 37 | F | Mid | 21 | Severe | 3/24/20 |
| 258 | 5 | 37 | No | 23 | M | Mid | 8 | Moderate | 3/26/20 |
| 277 | 13 | 45 | No | 65 | M | High | 16 | Moderate | 3/18/20 |
| 356 | 14 | 43 | No | 51 | F | Mid | 20 | Severe | 3/25/20 |
| 33 | 14 | 48 | No | 36 | M | Mid | 22 | Severe | 3/24/20 |
| 136 | 13 | 42 | Yes | 57 | F | High | 24 | Severe | 3/24/20 |
| 209 | 18 | 49 | No | 53 | F | Low | 14 | Moderate | 3/18/20 |
| 211 | 3 | 40 | No | 25 | F | Mid | 13 | Moderate | 3/23/20 |
| 261 | 15 | 43 | No | 63 | F | Low | 21 | Severe | 3/24/20 |
| 380 | 7 | 38 | No | 38 | F | Mid | 14 | Moderate | 3/25/20 |
| 219 | 15 | 45 | No | 27 | F | Mid | 21 | Severe | 4/3/20 |
| 279 | 19 | 48 | No | 32 | F | Mid | 19 | Severe | 3/21/20 |
| 293 | 17 | 63 | Yes | 72 | M | High | 22 | Severe | 3/8/20 |
| 305 | 4 | 47 | No | 43 | F | Mid | 14 | Moderate | 4/17/20 |
| 320 | 19 | 47 | No | 40 | F | Low | 19 | Severe | 3/17/20 |
| 326 | 15 | 47 | No | 36 | F | Mid | 18 | Moderate | 3/23/20 |
| 402 | 11 | 44 | No | 32 | F | Mid | 19 | Severe | 3/24/20 |
| 48 | 8 | 40 | No | 45 | F | High | 19 | Severe | 4/1/20 |
| 116 | 18 | 49 | No | 65 | F | Low | 13 | Moderate | 3/25/20 |
| 144 | 23 | 54 | No | 56 | M | Mid | 17 | Moderate | 3/16/20 |
| 210 | 7 | 41 | No | 47 | M | High | 16 | Moderate | 4/4/20 |
| 358 | 15 | 41 | No | 36 | F | Mid | 20 | Severe | 3/31/20 |
| 469 | 9 | 43 | Yes | 53 | F | High | 25 | Severe | 3/30/20 |
| 172 | 7 | 42 | No | 30 | F | Mid | 15 | Moderate | 3/25/20 |
| 468 | 16 | 54 | No | 22 | M | Mid | 11 | Moderate | 3/17/20 |
| 519 | 4 | 45 | No | 56 | F | Mid | 15 | Moderate | 3/25/20 |
| 535 | 10 | 48 | No | 30 | F | Low | 13 | Moderate | 3/23/20 |
| 423 | 5 | 38 | No | 58 | M | Mid | 4 | Mild | 4/8/20 |
| 20 | 19 | 48 | Yes | 31 | M | High | 29 | Critical | 3/31/20 |
| 564 | 32 | 60 | No | 24 | F | Low | 24 | Severe | 3/19/20 |
| 282 | 24 | 54 | No | 34 | F | High | 19 | Severe | 3/22/20 |
| 550 | 7 | 51 | No | 29 | M | Mid | 16 | Moderate | 3/19/20 |
| 127 | 16 | 46 | No | 57 | F | Low | 15 | Moderate | 4/22/20 |
| 175 | 17 | 49 | No | 54 | M | Mid | 16 | Moderate | 3/20/20 |
| 376 | 7 | 48 | No | 36 | F | Low | 8 | Mild | 3/31/20 |
| 201 | 18 | 58 | No | 56 | M | Mid | 20 | Severe | 3/16/20 |
| 437 | 6 | 35 | No | 28 | F | Low | 12 | Moderate | 4/5/20 |
| 573 | 17 | 56 | Yes | 25 | M | High | 25 | Severe | 3/20/20 |
| 17 | 17 | 55 | No | 42 | M | High | 20 | Severe | 3/21/20 |
| 586 | 17 | 61 | No | 32 | F | Mid | 18 | Moderate | 3/20/20 |
| 11 | 16 | 49 | Yes | 66 | M | High | 25 | Severe | 3/30/20 |
| 50 | 10 | 40 | No | 35 | M | Low | 13 | Moderate | 4/2/20 |
| 65 | 13 | 47 | No | 40 | F | Low | 16 | Moderate | 4/1/20 |
| 68 | 7 | 49 | No | 51 | M | Low | 6 | Mild | 4/2/20 |
| 348 | 14 | 48 | No | 29 | F | Low | 14 | Moderate | 3/26/20 |
| 407 | 11 | 43 | No | 34 | M | Mid | 16 | Moderate | 4/1/20 |
| 609 | 7 | 57 | No | 26 | F | Low | 16 | Moderate | 3/20/20 |
| 478 | 11 | 64 | No | 46 | F | Low | 16 | Moderate | 4/14/20 |
| 338 | 10 | 44 | No | 46 | M | Mid | 15 | Moderate | 4/4/20 |
| 214 | 24 | 59 | No | 47 | M | High | 20 | Severe | 3/26/20 |
| 622 | 11 | 46 | Yes | 40 | M | Low | 18 | Moderate | 4/5/20 |
| 223 | 0 | 73 | No | 80 | F | Low | 0 | Mild | 3/1/20 |
| 371 | 10 | 46 | No | 44 | M | Mid | 16 | Moderate | 3/29/20 |
| 451 | 11 | 49 | Yes | 46 | M | High | 26 | Severe | 4/4/20 |
| 537 | 14 | 59 | No | 36 | M | Mid | 13 | Moderate | 3/23/20 |
| 558 | 11 | 46 | No | 56 | F | High | 10 | Mild | 4/3/20 |
| 166 | 17 | 55 | No | 42 | F | Low | 18 | Moderate | 3/25/20 |
| 281 | 7 | 48 | No | 70 | M | Low | 9 | Mild | 3/29/20 |
| 447 | 21 | 61 | No | 42 | M | High | 24 | Severe | 4/1/20 |
| 626 | 19 | 56 | No | 44 | M | High | 14 | Moderate | 3/31/20 |
| 155 | 29 | 64 | No | 47 | F | Low | 20 | Severe | 3/19/20 |
