# Supplementary material for: SARS-CoV-2 Infection Severity Is Linked to Superior Humoral Immunity against the Spike
Source: mBio. 2021 Jan 19;12(1):e02940-20. doi: 10.1128/mBio.02940-20 (PMC7845638; doi:10.1128/mBio.02940-20)
Supplement: FIG S2 [file mBio.02940-20-sf002.docx]

**Extended data Fig. 2: Clinical data and antibody specificity of acutely infected subject clusters. a**-**c**, Age (**a**), sex (**b**), and CURB-65 score (**c**) of subjects in the high (n=15), mid (n=7), and low (n=13) responder clusters. **d**, Total Ig end point titers against ORF8 of subjects in the high (n=15), mid (n=7), and low (n=13) responder clusters. **e**, Proportion of subjects in the high (n=15), mid (n=7), and low (n=13) responder clusters with detectable antibodies (total Ig) against 1 or more NSP antigens. For **a** and **c**, data were analyzed by unpaired non-parametric Kruskal-Wallis tests. Data in **b** and **e** were analyzed by Fisher’s exact tests**.** Dashed lines in **d** are the limit of detection. Bars in **a** and **d** represent the median. Data in **a** and **d** are presented as the median with interquartile range.
