# Supplementary material for: SARS-CoV-2 Infection Severity Is Linked to Superior Humoral Immunity against the Spike
Source: mBio. 2021 Jan 19;12(1):e02940-20. doi: 10.1128/mBio.02940-20 (PMC7845638; doi:10.1128/mBio.02940-20)
Supplement: TABLE S3 [file mBio.02940-20-st003.docx]

**Supplemental Table 3**: ***P*-values between responder groups from the acutely infected cohort.** Related to Fig. 2. *P*-values of post-hoc pairwise comparisons of Analysis of Variance (ANOVA) between responder groups from the acutely infected cohort. *P* values were adjusted by Holm–Bonferroni method. *P* value = 0.00000 indicates *P* value < 0.00001. Red highlighted values represent statistically significant differences (*P ≤* 0.05) between groups.

|  | *P*-Values | | |  |
| --- | --- | --- | --- | --- |
| Antigen/Ab Isotype | High vs. Mid | Mid vs. Low | High vs. Low | Std. Deviation |
| NP IgG3 | 0.00000 | 0.00247 | 0.00000 | 1.65028627 |
| NP IgG | 0.00000 | 0.00000 | 0.00000 | 1.55238906 |
| NP IgA | 0.00000 | 0.00001 | 0.00000 | 1.39865608 |
| NP IgA1 | 0.00000 | 0.00010 | 0.00000 | 1.34647038 |
| NP IgA2 | 0.00000 | 0.00156 | 0.00000 | 1.25406493 |
| NP IgG1 | 0.00000 | 0.00000 | 0.00000 | 1.25071163 |
| NP IgG4 | 0.00067 | 0.63087 | 0.00003 | 1.24772617 |
| Spike IgG | 0.85674 | 0.00001 | 0.00000 | 1.12251522 |
| NP All Ab | 0.00270 | 0.00002 | 0.00000 | 1.11350163 |
| Spike All Ab | 0.03684 | 0.00004 | 0.00000 | 0.98688766 |
| Spike IgG3 | 0.53797 | 0.00046 | 0.00001 | 0.97972912 |
| Spike IgA | 0.28556 | 0.00266 | 0.00002 | 0.92272407 |
| ORF8 IgG | 0.41554 | 0.05648 | 0.00293 | 0.88124774 |
| Spike IgG1 | 0.04820 | 0.00023 | 0.00000 | 0.87884391 |
| NP IgM | 0.00002 | 0.19945 | 0.00000 | 0.87224077 |
| Spike IgA1 | 0.73044 | 0.00002 | 0.00000 | 0.86297411 |
| NP IgG2 | 0.00005 | 0.20110 | 0.00000 | 0.84540153 |
| Spike IgM | 0.22707 | 0.02034 | 0.11745 | 0.58655368 |
| NP IgD | 0.14321 | 0.37859 | 0.00964 | 0.5449614 |
| Spike IgA2 | 0.02472 | 0.00011 | 0.02472 | 0.51626759 |
| ORF8 All Ab | 0.37129 | 0.23313 | 0.01884 | 0.49777048 |
| ORF8 IgA | 0.39895 | 0.11318 | 0.00730 | 0.44250882 |
| Spike IgD | 0.86346 | 0.86346 | 0.37714 | 0.36506456 |
| Spike IgG4 | 0.41319 | 0.88793 | 0.32673 | 0.22016848 |
| ORF8 IgM | 0.94065 | 0.94065 | 0.48067 | 0.18630614 |
| ORF7a All Ab | 0.36390 | 1.00000 | 0.36390 | 0.09307027 |
| Spike IgG2 | 0.01960 | 0.45368 | 0.00092 | 0.01041245 |
