# Supplementary material for: SARS-CoV-2 Infection Severity Is Linked to Superior Humoral Immunity against the Spike
Source: mBio. 2021 Jan 19;12(1):e02940-20. doi: 10.1128/mBio.02940-20 (PMC7845638; doi:10.1128/mBio.02940-20)
Supplement: FIG S3 [file mBio.02940-20-sf003.docx]

**Extended data Fig. 3: Clinical data and antibody specificity of convalescent subject clusters. a**-**c**, Severity score (**a**), duration of symptoms (**b**), and days since symptom onset (**c**) of subjects in the high (n=23), mid (n=42), and low (n=40) responder clusters. **d** and **e**, Total Ig end point titers against N protein (**d**) and ORF8 (**e**) of subjects in the high (n=23), mid (n=42), and low (n=40) responder clusters. **f**, Proportion of subjects in the high (n=23), mid (n=42), and low (n=40) responder clusters with detectable antibodies (total Ig) against 1 or more NSP antigens. For **a**-**e**, data were analyzed by unpaired non-parametric Kruskal-Wallis tests. Data in **f** were analyzed by Fisher’s exact tests**.** Dashed lines in **d** and **e** are the limit of detection. Bars in **a**-**e** represent the median. Data in **a**-**e** are presented as the median with interquartile range.
