# Supplementary material for: SARS-CoV-2 Infection Severity Is Linked to Superior Humoral Immunity against the Spike
Source: mBio. 2021 Jan 19;12(1):e02940-20. doi: 10.1128/mBio.02940-20 (PMC7845638; doi:10.1128/mBio.02940-20)
Supplement: TABLE S4 [file mBio.02940-20-st004.docx]

**Supplemental Table 4**: ***P*-values between responder groups from the convalescent cohort.** Related to Figure 3. *P*-values of post-hoc pairwise comparisons of Analysis of Variance (ANOVA) between responder groups from the convalescent cohort. *P* values were adjusted by Holm–Bonferroni method. *P* value = 0.00000 indicates *P* value < 0.00001. Red highlighted values represent statistically significant differences (*P ≤* 0.05) between groups.

|  | *P*-Values | | |  |
| --- | --- | --- | --- | --- |
| Antigen/Ab Isotype | High vs. Mid | Mid vs. Low | High vs. Low | Std. Deviation |
| NP All Ab | 0.00000 | 0.00000 | 0.00000 | 0.97012491 |
| NP IgG | 0.00000 | 0.00000 | 0.00000 | 0.92822575 |
| NP IgG4 | 0.00006 | 0.29196 | 0.00000 | 0.65474305 |
| NP IgM | 0.44869 | 0.00063 | 0.00052 | 0.6466433 |
| ORF8 IgG | 0.00008 | 0.01025 | 0.00000 | 0.63588896 |
| NP IgG3 | 0.00211 | 0.00211 | 0.00000 | 0.63465636 |
| NP IgG1 | 0.00000 | 0.00000 | 0.00000 | 0.6337165 |
| Spike IgG3 | 0.00246 | 0.00246 | 0.00000 | 0.52020637 |
| Spike IgA1 | 0.46887 | 0.00000 | 0.00000 | 0.51098948 |
| Spike IgA | 0.52898 | 0.00000 | 0.00000 | 0.51020681 |
| Spike IgG1 | 0.00008 | 0.00001 | 0.00000 | 0.50629472 |
| NP IgA | 0.02180 | 0.00000 | 0.00000 | 0.50083625 |
| Spike IgG | 0.00027 | 0.00000 | 0.00000 | 0.49480188 |
| NP IgA1 | 0.03781 | 0.00000 | 0.00000 | 0.49265713 |
| Spike IgG4 | 0.35151 | 0.35151 | 0.11048 | 0.48422105 |
| Spike IgA2 | 0.72578 | 0.09653 | 0.28124 | 0.44303317 |
| Spike All Ab | 0.00021 | 0.00000 | 0.00000 | 0.39986788 |
| NP IgG2 | 0.00000 | 0.75031 | 0.00000 | 0.3439445 |
| Spike IgM | 0.98421 | 0.00668 | 0.01772 | 0.33589343 |
| ORF8 All Ab | 0.00024 | 0.01575 | 0.00000 | 0.31603601 |
| ORF8 IgM | 0.14538 | 0.14538 | 0.00870 | 0.28519883 |
| NP IgA2 | 0.73446 | 0.10013 | 0.10013 | 0.24871318 |
| Spike IgG2 | 0.70986 | 0.70986 | 0.38520 | 0.24461853 |
| ORF8 IgA | 0.00935 | 0.18456 | 0.00037 | 0.19149453 |
| Spike IgD | 0.80540 | 0.84585 | 0.80540 | 0.15863626 |
| NP IgD | 1.00000 | 1.00000 | 1.00000 | 0.05778103 |
| ORF7a All Ab | 0.81966 | 0.81966 | 1.00000 | 0.01082894 |
