# Supplementary material for: SARS-CoV-2 Infection Severity Is Linked to Superior Humoral Immunity against the Spike
Source: mBio. 2021 Jan 19;12(1):e02940-20. doi: 10.1128/mBio.02940-20 (PMC7845638; doi:10.1128/mBio.02940-20)
Supplement: TABLE S5 [file mBio.02940-20-st005.docx]

**Supplemental Table 5: Infection severity scoring system for convalescent subjects based on symptoms, hospitalization, and duration of symptoms.**

| **Symptom** | **Severity** | **Score** |
| --- | --- | --- |
| Fatigue | Moderate | 2 |
| Cough | Mild | 1 |
| Shortness of Breath | Moderate | 2 |
| Congestion | Mild | 1 |
| Sore Throat | Mild | 1 |
| Fever | Moderate | 2 |
| Headache | Mild | 1 |
| Myalgia | Moderate | 2 |
| Abdominal Pain | Mild | 1 |
| Diarrhea | Moderate | 2 |
| Loss of Taste | Mild | 1 |
| Loss of Smell | Mild | 1 |
| **Hospitalization** | **Score** | |
| No | 0 | |
| Yes, without Oxygen | 6 | |
| Yes, with Oxygen | 8 | |
| Yes, ICU Admission | 10 | |
| **Duration of Symptoms** | **Score** | |
| 0 days | 0 | |
| < 7 days | 2 | |
| 7-13 days | 4 | |
| 14-20 days | 6 | |
| 21+ days | 8 | |
| **Infection Severity** | **Cumulative Score** | |
| Mild Infection | ≤ 10 | |
| Moderate Infection | 11-18 | |
| Severe Infection | 19-27 | |
| Critical Infection | 28-35 | |
