# Supplementary material for: SARS-CoV-2 Infection Severity Is Linked to Superior Humoral Immunity against the Spike
Source: mBio. 2021 Jan 19;12(1):e02940-20. doi: 10.1128/mBio.02940-20 (PMC7845638; doi:10.1128/mBio.02940-20)
Supplement: FIG S4 [file mBio.02940-20-sf004.docx]

**Extended data Fig. 4: MBC responses against N protein and ORF8. a** and **b**, PBMCs from convalescent donors were polyclonally stimulated, and ELISPOTs were performed to assess the number of antigen-specific MBCs. **a**, Number of IgG/IgA^+^ MBCs (antigen-specific MBCs per 10^6^ cells) targeting N protein (**a**) and ORF8 (**b**) amongst the high (n=14 for N protein; n=11 for ORF8), mid (n=16 for N protein; n=15 for ORF8), and low responder (n=10 for N protein and ORF8) clusters**.** For **a** and **b**, data were analyzed by unpaired non-parametric Kruskal-Wallis tests. Data in are presented as the median with interquartile range.
